# Ultrasound and clinical outcomes of vascularized ulnar nerve transposition

**DOI:** 10.1186/s13018-026-06891-4

**Published:** 2026-05-04

**Authors:** Ruben Dukan, Remy Pichard, Pascal Jehanno, Valerie Vuillemin, Benjamin Morino, Maxime Lacroix

**Affiliations:** 1 Hand Surgery Department, HEGP, Paris, France; 2 Radiologic Department, HEGP, Paris, France; 3 Clinique de la Main de Paris, 36 bis rue nicolo, 75116 Paris, France

**Keywords:** Ulnar nerve, Entrapment, Vascularized, Transposition

## Abstract

**Background:**

Ulnar nerve entrapment at the elbow is common but results of ulnar nerve transposition remained uncertain. Chronic nerve ischemia is implicated in the syndrome’s pathophysiology, and surgical interventions aim to mitigate related damage. The main hypothesis is that preserving the vascular system during ulnar nerve transposition helps prevent ischemic damage and improves clinical outcomes.

**Methods:**

Thirty five patients were included and divided into two groups: ‘Vascularized Ulnar Nerve’ (UNV+) and ‘Non-vascularized Ulnar Nerve’ (UNV–). They underwent subcutaneous anterior transposition of the ulnar nerve. Preoperative and postoperative clinical assessment was made using: Bishop score, Visual Analog Scale, Levine-Katz survey, Functional Status Scale, and QuickDash Score. Post operative nerve extrinsic vascularization was assessed with ultrasound.

**Results:**

Results after a mean 19.2-month follow-up show superior clinical outcomes, including Bishop and Levine-Katz scores, in the ‘Vascularized Ulnar Nerve’ group. Despite longer operative times (47 min in UNV + vs. 33 min in UNV–), no vascular complications occurred, supporting the hypothesis that preserving extrinsic vascularization enhances results. Extrinsic vascularization of the ulnar nerve could be continuously observed from proximal to distal in all patients in the UNV+ group. In 4 out of 15 cases in the UNV– group, vascularization was observed along the entire length of the nerve.

**Conclusions:**

Ultrasound assessments indicated improved nerve characteristics in the vascularized ulnar nerve group, reinforcing the study’s main conclusion: preserving the extrinsic vascular system during ulnar nerve transposition was associated to better clinical outcomes. However, the study acknowledges limitations, urging further research with larger cohorts and electromyographic evaluation for a comprehensive understanding.

**Level of evidence IV:**

Case series Therapeutic study.

**Supplementary Information:**

The online version contains supplementary material available at 10.1186/s13018-026-06891-4.

## Introduction

Ulnar nerve entrapment at the elbow corresponds to the most common entrapment neuropathy after carpal tunnel syndrome [1]. Various surgical techniques have been described, ranging from in situ deentrapment under endoscopy to anterior transposition, whether subcutaneous or submuscular.

The pathophysiology of ulnar nerve entrapment at the elbow is multifactorial, and one of the factors should be chronic nerve ischemia [2]. Although instability represents a dynamic mechanical phenomenon distinct from static compression, repeated subluxation during elbow flexion may induce repetitive traction and intermittent vascular compromise, potentially contributing to chronic microvascular impairment. This mechanism may justify anterior transposition in cases of documented instability. Transposition surgery is conceptually designed to alleviate the tension that lead to neural ischemia, demyelination, and axon loss in cubital tunnel syndrome. Therefore, it is important to preserve extrinsic nerve vascularization during the surgical procedure. These vessels connect to the nerve via the fascia and anastomose with the intrinsic system of the nerve. The intrinsic system is composed of all the vessels within the epineural sheath. In general, the nutritional supply of the nerve is essential for normal nerve conduction and axonal transport. Therefore, restoration of intraneural blood flow is critical for the recovery of peripheral nerve function following chronic entrapment [3]. Felder [4] reported that improperly performed Ulnar Nerve Transposition (UNT) can cause significant patient morbidity with pain and neuropathy. The technique of nerve and vascular network transposition was described by Messina in 1996 [5]. The author’s main hypothesis was that chronic ulnar nerve entrapment resulted in a decrease of extrinsic and intrinsic vascularity. However, that study was retrospective and lacked a control group, limiting internal validity and potentially overestimating treatment effect. Although the author reported satisfactory results, Nakamura [6] found no impact of the preservation of vascularization on clinical outcomes at one year of follow-up. However, the main limitation lies in the measurement of vascular preservation using Doppler sonar immediately postoperatively and not remotely, at the same follow-up interval as the clinical outcomes. This refutes Ogata’s theory [7] that showed anterior transposition is associated with a significant decrease in the extrinsic arterial supply, especially just distal to the groove for at least three days.

Recently, Yeoh [8] also reported that damage to the extrinsic and intrinsic vascular systems of a nerve can result from excessive mobilization. The critical length of mobilization is 7 cm in cases of impairment of both vascular networks. Felder, in the context of ulnar nerve transposition, specifies that nerve release should occur over at least 6–8 cm [4], corresponding to impairment of both extrinsic and intrinsic systems unless these are preserved.

The main hypothesis is that preserving the extrinsic vascular system during ulnar nerve transposition helps prevent ischemic damage and improves clinical outcomes.

## Material and methods

This single center study, nonrandomized, retrospective study, conducted from January 2020 to November 2022, included 35 patients who underwent subcutaneous anterior transposition of the ulnar nerve. In one group, practitioners (RD and RP) performed the intervention with special attention to the dissection and preservation of the extrinsic vascular pedicle. The patients were divided into two groups: ‘Vascularized Ulnar Nerve’ (UNV+) and ‘Non-vascularized Ulnar Nerve’ (UNV–).

The inclusion criteria were: (1) Primary cubital ulnar tunnel syndrome (tinel sign at the cubital tunnel) with failed conservative management of at least three months, including activity modification, night splinting, and non-steroidal anti-inflammatory drugs when tolerated, (2) sonographic confirmation of ulnar nerve instability: subluxation or true dislocation, (3) follow-up of more than 1 year. All included patients presented with documented dynamic ulnar nerve instability (subluxation or dislocation) confirmed by ultrasound. In our practice, the presence of dynamic instability is considered an indication for anterior transposition rather than isolated in situ decompression, as decompression alone does not address the mechanical translation of the nerve during elbow flexion–extension. Therefore, simple decompression was not performed in this cohort. Electroneuromyographic confirmation was mandatory to confirm the surgical indication.

The exclusion criteria were: (1) age < 18 years old, (2) history of ulnar nerve deentrapment, (3) post-fracture or elbow dislocation, (4) neuromuscular disease or diabetes.

Informed consent was obtained from each patient and study was approved by the local institutional board. The study was conducted in compliance with the principles of the Declaration of Helsinki and its later amendments. All patients were informed that their anonymized data could be used for research purposes, and none expressed opposition.

### Surgical technique

The intervention was performed under axillar block anesthesia, in a supine position, and using magnifying loupes.

A curved skin incision approximately 8 cm long was made posterior to the medial epicondyle. The medial antebrachial cutaneous nerve was preserved. The ulnar nerve was identified proximally, and the arcade of Struthers and medial intermuscular septum were incised. Subsequently, the cubital tunnel retinaculum was opened, along with the fascia of the flexor carpi ulnaris and the Osborne ligament. The proximal motor branch for the flexor carpi ulnaris (FCU) was dissected to allow for transposition.

In the unvascularized group, only the intrinsic system in the vasa nervorum was preserved and the extrinsic supply was not transposed.

**Fig. 1:**
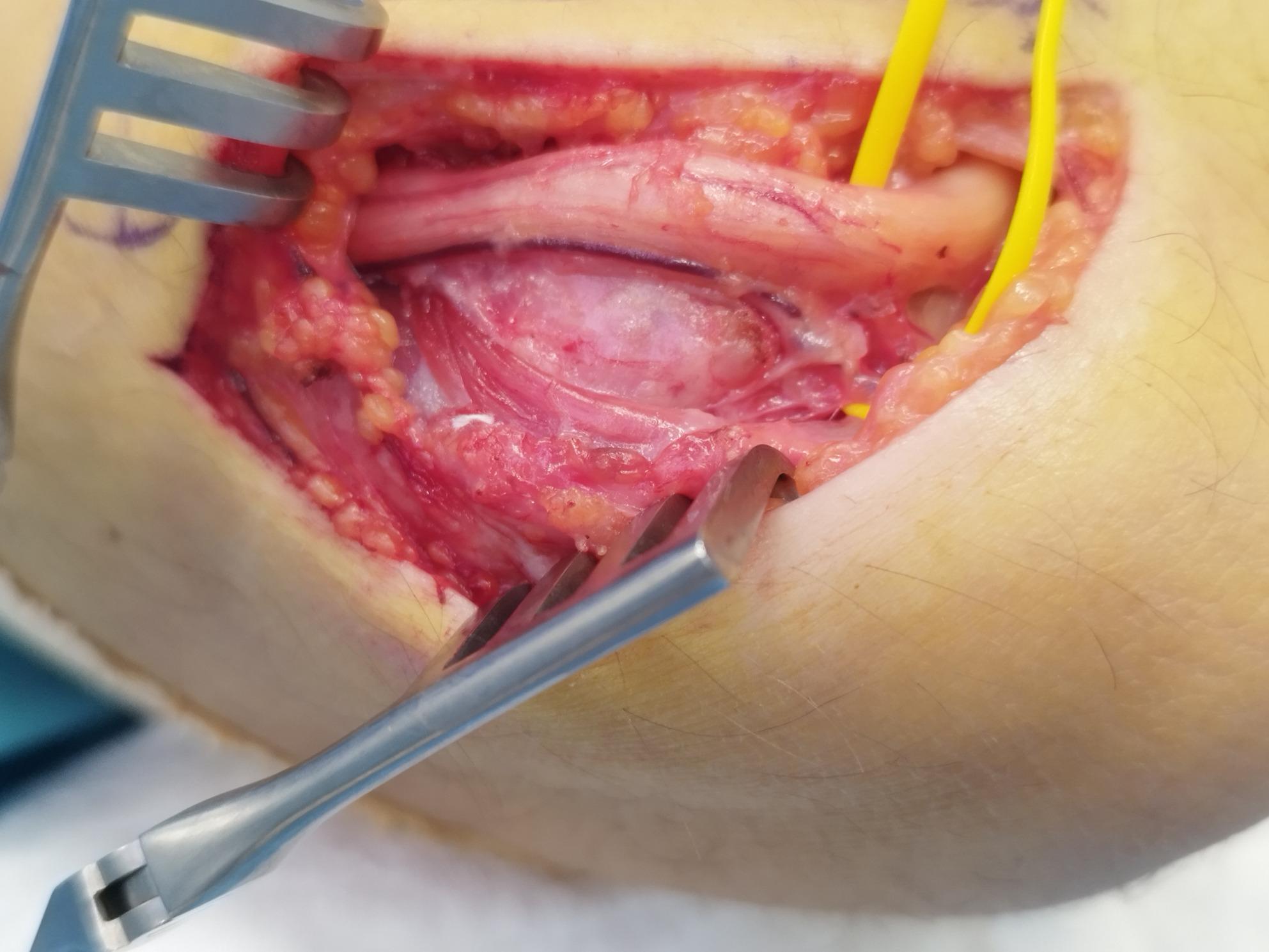
Intraoperative picture illustrating identification and preservation of the extrinsic vascular arcade

In the vascularized group, interventions were performed without a tourniquet. The extrinsic vascular axis was carefully identified and dissected from proximal to distal. Only minor posterior collateral branches were selectively coagulated to allow mobilization of the vascular arcade, while preserving the main longitudinal vascular axis supplying the nerve. Given the extensive anastomotic network between the superior and inferior ulnar collateral systems, selective interruption of small posterior branches does not compromise overall nerve perfusion. The integrity of the preserved vascular axis was verified intraoperatively by visual inspection (Fig. 1). At the end of the intervention, the operator ensured that the performed release allowed for the anterior displacement of the nerve and its vascularization without tension or kinking. The harvesting of the adipofascial flap was done, and the flap was fixed to the epicondyle with 3.0 PDS (resorbable) sutures, ensuring a sufficient tunnel to avoid entrapment of the nerve or its vascularization and preventing any entrapment effect during elbow flexion-extension movements.

### Post operative protocol

Postoperatively, a thick dressing was kept in place for one week. No physical therapy was required. The patient was allowed to freely mobilize their elbow.

### Clinical assessment

The MacGowan score was used to classify the severity of the pathology preoperatively. Preoperative symptoms (noted as present or absent) fell into four types: paresthesias, loss of sensation, loss of strength, and motor deficit. These symptoms were reassessed in the same manner during various postoperative evaluations.

The primary outcome was the Bishop score. Secondary outcomes included elbow range of motion in degrees (measured with a goniometer), scar pain assessed using the Visual Analog Scale (VAS) from 0 to 10, Levine-Katz survey, Functional Status Scale, and QuickDash Score. Questionnaires, including the Levine-Katz survey, were administered to subjects by phone or in the office to assess long-term relief of ulnar symptoms and satisfaction with the procedure.

An independent observer clinically followed patients at regular intervals, from the preoperative period until the last follow-up. Perioperative and postoperative complications were documented.

### Ultrasound Doppler assessment

Two radiologists (ML and BM), each with six years of experience in musculoskeletal imaging, conducted elbow ultrasound examinations for all patients. Each patient underwent assessments before the surgical procedure and one month afterward, following a double-blind approach. Both radiologists were unaware of the patient’s assigned arm and the decision regarding vascular preservation. Additionally, neither radiologist had access to the patient’s medical records, including the ultrasound performed by the other one. Consequently, each patient underwent four examinations (two before and two after surgery), by two different radiologists.

**Fig. 2:**
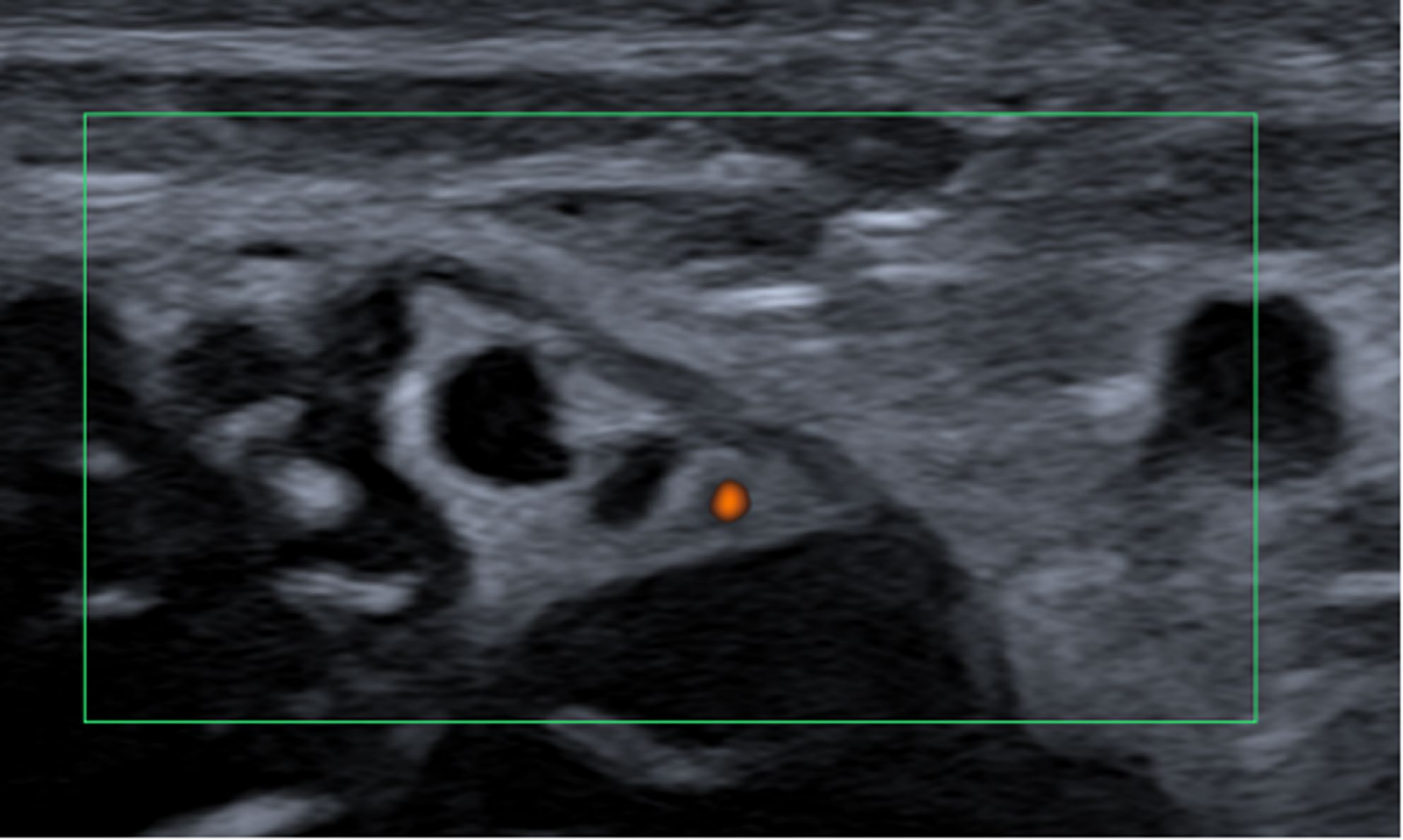
Axial section of power Doppler ultrasound, highlighting arterial vascularization adjacent to the ulnar nerve

A Canon Aplio i800 system (Canon Medical System, USA) with a 33 MHz high-frequency matrix linear transducer (i33LX9) was used. The evaluation of morphological criteria for ulnar nerve entrapment on B-mode imaging included measuring the cross-sectional area of the ulnar nerve in the transverse plane (Fig. 2). This measurement was taken within the inner border of the hyperechoic epineurium using the free-hand tracer tool. Additionally, echogenicity of the nerve, entrapment zone characteristics, stability during dynamic maneuvers, and the identification of anatomical variants, such as the anconeus epitrochlearis muscle, were considered in the assessment.

**Fig. 3:**
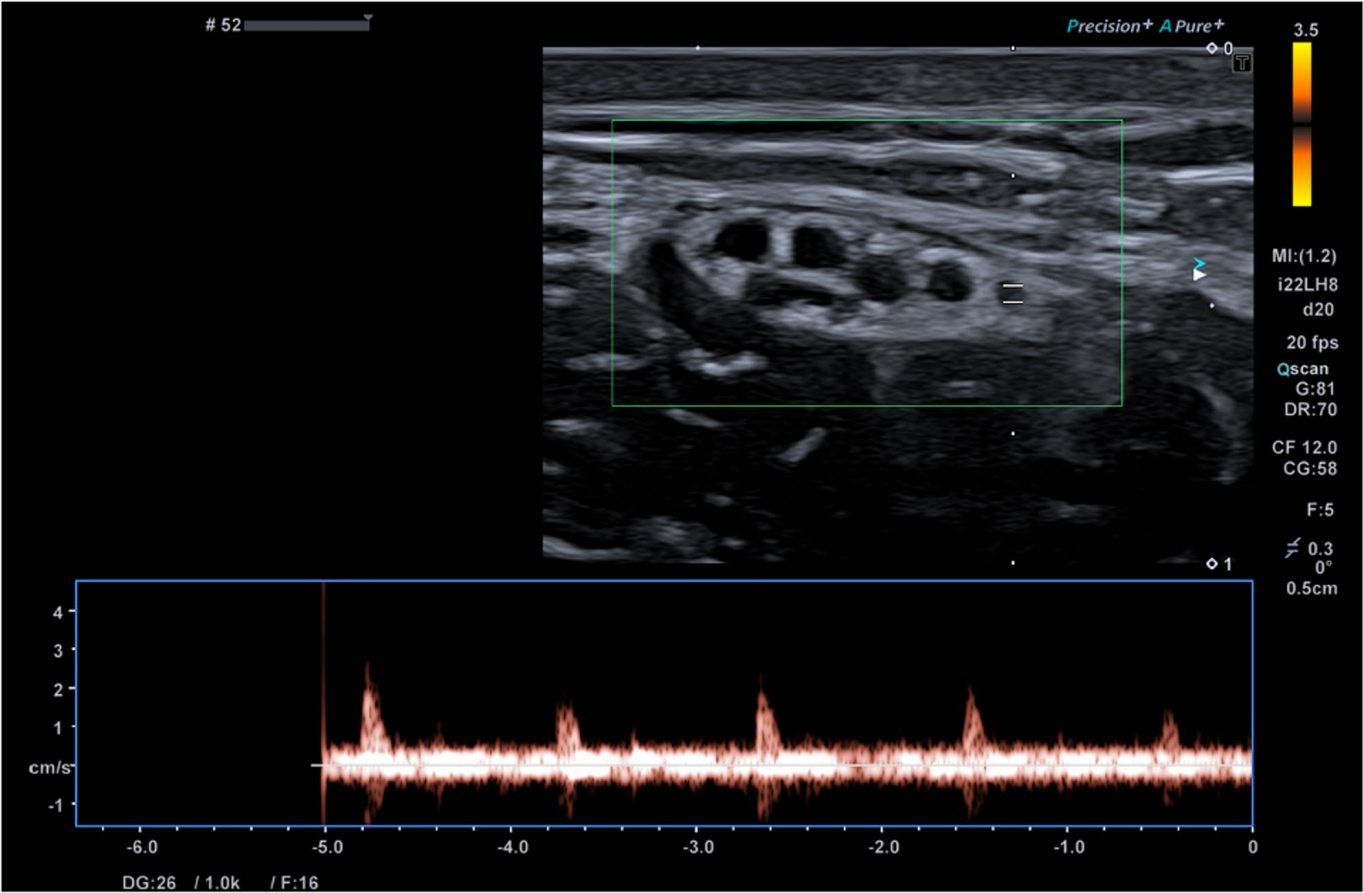
Axial section of pulsed wave Doppler ultrasound as a noninvasive method for estimating pulse wave velocity of the ulnar arterial vascularization

The determination of the presence or absence of a visible artery was evaluated using power Doppler and superb microvascular imaging (SMI). In cases when an artery was identified, the assessment of blood flow to the ulnar nerve was conducted at three specific locations: within the region of the arcade of Struthers (superior ulnar collateral artery), in the cubital groove (inferior ulnar collateral artery), and between the two heads of the flexor carpi ulnaris, known (posterior ulnar recurrent artery). Pulsed Doppler was systematically measured to confirm the arterial nature (triphasic waveform) (Fig. 3). Vascularization assessment was qualitative and based on the presence or absence of a detectable arterial signal confirmed by triphasic waveform analysis. No quantitative Doppler flow thresholds or standardized perfusion values were predefined in this study.

### Statiscal analysis

Data were analyzed using SAS 9.1 software (SAS Institute, Cary, IN, USA).

Given the retrospective design and limited sample size, statistical analysis was primarily descriptive and comparative. Qualitative variables were analyzed using Fisher’s exact test. Continuous variables were compared using Student’s *t*-test, as this test is relatively robust to moderate deviations from normality in small independent samples.

The Bishop score was defined as the primary outcome measure of the study. Levine–Katz and QuickDASH scores were considered secondary outcomes. Other clinical and ultrasonographic variables were regarded as exploratory.

A multivariable linear regression model was performed with the Bishop score as the dependent variable, adjusting for MacGowan stage, motor weakness, smoking status, and age. Sensitivity analyses included nonparametric testing using the Mann–Whitney U test and stratified analysis restricted to stage 2 A patients.

Statistical significance was set at *p* < 0.05.

## Results

Sixteen patients were included in the ‘Vascularized Ulnar Nerve’ group (UNV+), and 19 in the ‘Non-vascularized Ulnar Nerve’ group (UNV–). Two patients were lost to follow-up in the UN-V+ group, and four were lost to follow-up in the UNV– group. In total, 14 patients were analyzed in the UNV+ group, and 15 patients in the UNV– group.

The demographic criteria were similar in both groups (Table 1). The mean age was 44 years (SD 14) in the UNV+ group versus 52 years (SD 16) in the UNV– group. In 6 cases in the UNV+ group, patients were manual laborers, compared to 2 cases in the UNV– group.

**Table 1:** Demographic data

	UNV+ *N* = 14	UNV– *N* = 15
Sex (H/F)	9/5	7/8
Age	44.2 (SD 13.8)	52.2 (SD 16.4)
Smoking status	0	2
Diabetes	1	1
Manual worker	6/14	2/15
Work accident	1/14	2/15

Preoperatively, the 14 patients in the UNV+ group were classified as stage 2 A according to the MacGowan score. In the UNV– group, 1 patient was classified as stage 1, 10 as stage 2 A, 2 as stage 2B, and 2 as stage 3.

The electromyoneurography (ENMG) was positive in all cases, and instability was confirmed by ultrasound.

### Clinical results

At the end of the average follow-up of 19.2 months (SD 8.9), the MacGowan score was 0 in 11 out of 14 cases and grade I in 3 out of 14 cases in the UNV+ group. In the UNV– group, patients gained an average of one grade in the MacGowan classification.

The Bishop score was 10 (SD 1) in the UNV+ group versus 6.2 (SD 2.6) in the UNV– group (*p* < 0.001). The Levine-Katz score was significantly lower in the UNV+ group (15.8 (SD 6.3) vs. 30.1 (SD 12.2), *p* < 0.001). The Functional Status Scale showed that most patients in the UNV+ group had no difficulty or slight difficulty in performing various tasks, while patients in the UNV– group generally experienced slight or moderate difficulty (Table 2). Manual laborers in the UNV+ group were able to return to their previous jobs, while one of the two manual laborers in the UNV– group required job reassignment.

**Table 2:** Clinical results

	UNV+ *N* = 14	UNV– *N* = 15	*p*
Follow-up	19.3 (SD 8.3)	19.2 (SD 9.7)	
Preoperative ulnar paresthesia	14/14	15/15	n.a
Paresthesia improved	3/14	5/15	
Paresthesia resolved	11/14	5/15	
Preoperative loss of strength	14/14	12/15	n.a
Improvement	8/14	2/15	
Preoperative motor weakness	0	4/15	n.a
Improvement	0	2/15	
Patient satisfaction			
Would undergo same operation again	14/14	11/15	0.09
Would recommend operation	14/14	10/15	0.04
Scar VAS	1.2 (SD 1.4)	2.6 (SD 1.8)	0.11
Bishop score	10 (SD 1)	6.2 (SD 2.6)	< 0.001
Levine Katz	15.8 (SD 6.4)	30.1 (12.2)	< 0.001
Functional status scale	1.5 (SD 0.4)	2.5 (SD 1)	0.001
QuickDash score	15.6 (SD 14.9)	57.4 (24.9)	< 0.001

No perioperative or postoperative complications were identified in either group at the end of the follow-up.

To account for baseline differences between groups, a multivariable linear regression analysis was performed with the Bishop score as the dependent variable, adjusting for MacGowan stage, motor weakness, smoking status, and age (Table 3).

**Table 3:** Adjusted analysis of clinical outcomes

Outcome	Adjusted mean difference (UNV + vs. UNV−)	95% CI	*p*-value
Bishop score (primary outcome)	+ 2.1	0.9–3.3	0.001
Levine–Katz score	−0.42	−0.72 to −0.12	0.008
QuickDASH score	−10.6	−17.8 to −3.4	0.005

Model adjustment variables:

MacGowan stage, motor weakness, smoking status, age

After adjustment, vascularized anterior transposition remained significantly associated with higher Bishop scores compared with nonvascularized transposition (adjusted mean difference 2.1 points, 95% CI 0.9–3.3, *p* = 0.001).

Secondary outcomes showed similar associations. Levine–Katz scores were significantly lower in the vascularized group (mean difference − 0.42, 95% CI − 0.72 to − 0.12, *p* = 0.008), as were QuickDASH scores (mean difference − 10.6, 95% CI − 17.8 to − 3.4, *p* = 0.005).

Nonparametric sensitivity testing using the Mann–Whitney U test confirmed the robustness of these findings (Bishop *p* = 0.002, Levine–Katz *p* = 0.01, QuickDASH *p* = 0.01).

In addition, a stratified sensitivity analysis restricted to patients with MacGowan stage 2 A demonstrated consistent results, with higher Bishop scores observed in the vascularized group (mean difference 1.8, 95% CI 0.4–3.2, *p* = 0.02).

### Ultrasound results

Preoperatively, instability was observed in all patients in both groups.

Postoperatively, the nerve was significantly thicker in the UNV– group (*p* = 0.03), with 12 out of 15 cases showing a hypoechoic nerve and loss of fibrillar appearance. No residual entrapment was reported in the UNV+ group, while one case of residual entrapment at the flexor carpi ulnaris (FCU) was reported in the UNV– group. No further therapeutic intervention was performed in this patient at the last follow-up because, although symptomatic (paresthesia), the patient declined surgical revision.

Extrinsic vascularization of the ulnar nerve could be continuously observed from proximal to distal in all patients in the UNV+ group. In 4 out of 15 cases in the UNV– group, vascularization was observed along the entire length of the nerve (Table 4).

**Table 4:** Postoperative ultrasound findings

	UNV+ *N* = 14	UNV– *N* = 15	*p*
Thick ulnar nerve	5/14	11/15	0.03
Hypoechogenous aspect	6/14	12/15	0.06
Hyperemia doppler	0	0	n.a
Residual entrapment	0	1/15	
Instability	0	0	n.a
Artery visualization	14/14	4/15	< 0.001

## Discussion

We have reported the comparative results of anterior transposition of the ulnar nerve with preservation of the extrinsic vascular arcade. The clinical results reported at the end of the follow-up show a significant superiority in the UNV+ group compared to the UNV– group.

This confirms Yeoh’s theory [8]. Yeoh reported that excessive mobilization of an intact peripheral nerve may compromise both extrinsic and intrinsic vascular networks. In their experimental model, mobilization exceeding approximately 7 cm was associated with impairment of vascular perfusion. This value refers specifically to mobilization in intact nerves and should not be interpreted as a general critical length for all vascularized nerve segments.

Therefore, preserving the vascular pedicles of the ulnar nerve during subcutaneous anterior transposition could have the advantage of better clinical results; however, it has disadvantages such as longer operative time, the need to perform careful dissection, and the risk of further vascular damage. In this series, the operative time was significantly increased in the UNV+ group, with a duration approximately 1.5 times longer (47 min in UNV + vs. 33 min in UNV–). However, no vascular complications were reported. Danoff [1], reported that the most frequent postoperative complication is hematoma.

From an anatomical perspective, Frantz [9] explains that in its native posterior position in the retrocondylar groove, the ulnar nerve is subject not only to entrapment forces at multiple sites but also to traction forces that increase intra-neural pressure [10] and potentially compromise nerve microcirculation as the elbow is flexed. The extrinsic vascular system is composed of three arteries: the superior ulnar collateral artery, the inferior ulnar collateral artery, and the posterior ulnar recurrent artery [2]. Multiple anastomoses exist between these different arteries. The main axis and anastomoses are mainly located in the posterolateral part, so vascular dissection should be performed, after identifying the nerve, from proximal to distal, releasing the superomedial segment. This area is considered the safe vascular zone. The posterior collaterals are numerous and should be ligated or electrocoagulated to allow mobilization. Connections between the extrinsic and intrinsic systems are numerous, and lesions on one of the anastomoses will not result in ischemic damage. Zochodne in 2017 [11], reported that the endoneurial blood flow is less prone to damage from interrupting single supplying vessels.

Thus, we believe that the disappointing results of anterior transpositions of the ulnar nerve may be partly due to nerve ischemia caused by surgery. Filippi and colleagues [12] reported that patients requiring reoperation after primary anterior transposition presented significant epineural and perineural fibrosis. Although vascular compromise could theoretically contribute to postoperative tissue changes, a direct causal relationship between postoperative nerve ischemia and perineural fibrosis has not been demonstrated and remains speculative. On the other hand, in four cases of the UNV– group, continuous vascularization was found on Doppler ultrasound at the last follow-up. Persistent vascularization in these cases may reflect either partial preservation of the vascular arcade during surgery or secondary neovascularization occurring during postoperative healing. Given that Doppler assessment was qualitative, differentiation between these mechanisms is not possible. These findings should therefore be interpreted cautiously. The clinical results of these patients are similar to the UNV+ group at the last follow-up. However, patients in this group reported persistent postoperative pain in the first 3 months, which may correspond to a phase of hypovascularization followed by revascularization.

We believe that the main difference between in situ deentrapment techniques and anterior transposition techniques is the preservation of vascularization. Importantly, all patients included in this study had ultrasound-confirmed dynamic instability. In this specific setting, anterior transposition was selected to treat both entrapment neuropathy and mechanical instability. Therefore, results from simple in situ decompression studies are not directly comparable to our cohort, as many comparative series exclude unstable nerves or allocate instability cases to transposition. Studies by Byvaltsev [13] and Buchanan [14], found no difference between open and endoscopic techniques. The surgical technique is independent of preoperative clinical severity [15]. Bonczar et al. [16] conducted a meta-analysis comparing single deentrapment versus submuscular transposition. Simple deentrapment is associated with a higher frequency of overall improvement compared to anterior transposition. Subcutaneous transposition trended higher than simple deentrapment in overall complication, wound infection, sensibility loss, nerve injury, hematoma, elbow or scar pain, and paresthesia or tenderness. This supports the theory of the impact of preserving vascularization on clinical outcomes [13].

The study by Geutjens [17] is very interesting because in situations of instability, some clinicians prefer epicondylectomy over anterior transposition. The author compared medial epicondylectomy and ulnar nerve transposition for CubTS in a prospective randomized study and found medial epicondylectomy to be more satisfactory. The author partially explained these results by stating that one major criticism associated with the anterior transposition procedure is that it devascularizes larger fragments of the nerve. Said [15] partly confirmed these results in 2018 in a study comparing simple deentrapment with or without medial epicondylectomy and ulnar nerve transposition, either in subcutaneous transposition or intra- and submuscular transposition. No clinical differences could be observed due to the significant heterogeneity of the groups. However, patients in the transposition groups presented more postoperative complications. The author explains this by the larger incision, more nerve manipulation, and wider dissection required for transposition.

This study has several limitations. It involves a small cohort with a limited number of patients. A comparative study with a larger cohort is necessary. baseline differences were observed between groups, including greater disease severity according to MacGowan classification, presence of motor weakness, and smoking in the UNV− group. As this study was not randomized, these differences may have influenced postoperative outcomes. Therefore, a direct causal relationship between vascular preservation and improved clinical results cannot be definitively established, and the findings should be interpreted as hypothesis-generating. The long-term evaluation was solely clinical, with an ultrasound examination to confirm the correct preservation of the extrinsic system. In addition, Doppler assessment in this study was qualitative rather than quantitative. Although the presence of a triphasic arterial signal confirms vascular flow, no standardized Doppler reference values were used to define normal versus impaired perfusion. Future studies should aim to establish quantitative Doppler parameters to better characterize postoperative nerve vascularization. Electromyographic evaluation could be considered to assess whether vascular preservation has an impact on electromyographic results. There was no preoperative evaluation of the fibrillar aspect and caliber of the nerve, nor a reference for vascularization. We believe that this vascularization is always present. However, it would have been interesting to know whether nerve hypertrophy and the loss of fibrillar characteristics observed in the UNV– group were present preoperatively or if they correspond to the consequences of iatrogenic nerve ischemia. Finally, the difference in results observed in this series may be due to the greater severity of patients in the UNV– group, according to the MacGowan classification. Multiple outcomes were analyzed without formal correction for multiplicity. Therefore, *p*-values close to the significance threshold should be interpreted cautiously, and the findings should be considered hypothesis-generating. As no correction for multiple comparisons was applied, secondary and exploratory outcome findings should be interpreted with caution and considered hypothesis-generating. Our findings apply specifically to patients with cubital tunnel syndrome associated with dynamic instability and should not be generalized to patients suitable for simple decompression.

## Supplementary Information

Below is the link to the electronic supplementary material.

Supplementary Material 1.

## Data Availability

The datasets generated and/or analysed during the current study are not publicly available due to patient confidentiality and local data protection regulations but are available from the corresponding author on reasonable request.
