# Supplementary material for: Ultrasound and clinical outcomes of vascularized ulnar nerve transposition
Source: J Orthop Surg Res. 2026 May 4;21:389. doi: 10.1186/s13018-026-06891-4 (PMC13359414; doi:10.1186/s13018-026-06891-4)
Supplement: Supplementary file 1 — Supplementary Material 1. [file 13018_2026_6891_MOESM1_ESM.docx]

Dear Editor,

Here it is the manuscript  entitled : **Ultrasound-Clinical outcomes of Vascularized ulnar nerve transposition.** This manuscript was approved by all the authors. We believe that the manuscript represents honest work.

Sincerely

Dr Dukan
